# Supplementary material for: Knowledge and Attitude Regarding Monkeypox Virus among Physicians in Saudi Arabia: A Cross-Sectional Study
Source: Vaccines (Basel). 2022 Dec 8;10(12):2099. doi: 10.3390/vaccines10122099 (PMC9781185; doi:10.3390/vaccines10122099)
Supplement: Supplementary file 1 [file vaccines-10-02099-s001.zip › vaccines-2008818-supplementary.pdf]

Knowledge and Attitudes of Monkeypox viral infection among Physicians in Saudi Arabia.

Survey

Consent question: -

This survey aims to assess the knowledge about Monkeypox among Saudi physicians. You are then kindly invited to answer the following online survey (on your computer or your mobile phone). We would like to kindly ask you to do your best to answer all questions, if possible, because this will allow us to anonymously process and analyze the data, and thus, improve the knowledge in the field.

- By ticking in the following case, you acknowledge the following:

  - I am Saudi;
  - I am a Physician;
  - I have read the content of this document including the purpose of the survey/questionnaire;
  - I agree to answer, as accurately as possible, the questions of the Survey/ Questionnaire;
  - I freely consent to participate to this study.

- **Socio-demographics questions:**
- Age in years:
- Gender ✓ Male ✓ Female.
- Marital status ✓ Married ✓ Single.
- -Level of work: Intern ✓GP✓ Resident ✓ Registrar✓ Senior registrar ✓ Consultant.
- -Your medical specialty: Medical Intern, General (GP), Internal medicine, Family medicine, Preventive medicine, Pediatrics, Dermatology, Emergency medicine, Other.
- -The institution you Work in: Governmental, Private.
- -Medical practice experience: less than 1 year, 1-5 years, More than 5 years.
- Which region of Saudi Arabia you are from? ✓ Central ✓ Western ✓ Northern ✓ Southern ✓ Eastern.
- -Had you ever received information of human Monkeypox during medical education: yes, No.
- -Had you ever heard about human Monkeypox before: Yes, No.
- When your first time you heard information about Monkeypox: Within several days or weeks ago, within last month or later, I did not hear about it.
- **Knowledge about Monkeypox questions:**
- Monkeypox is prevalent in middle eastern countries: Yes, No.
- Monkeypox is prevalent in Western and Central Africa:
- Yes, No.
- -There are many human monkeypox cases in Saudi Arabia: Yes, No.

- Monkeypox is a viral disease infection: Yes, No.
- -Monkeypox is a bacterial disease infection: Yes, No.
- -Monkeypox is easily transmitted human-to-human: Yes, No.
- -Monkeypox could be transmitted through a bite of an infected monkey: Yes, No
- -Travelers from America and Europe are the main source of imported cases of monkeypox: Yes, No.
- -Monkeypox and smallpox have similar signs and symptoms: Yes, No.
- -Monkeypox and chickenpox have similar signs and symptoms: Yes, No
- Flu-like syndrome is one of the early signs or symptoms of human monkeypox: Yes,No
- Rashes on the skin are one of the signs or symptoms of human monkeypox: Yes, No
- -Papules on the skin are one of the signs or symptoms of human monkeypox: Yes, No
- Vesicles on the skin are one of the signs or symptoms of human monkeypox: Yes, No.
- -Pustules on he skin are one of the signs or symptoms of human monkeypox: Yes, No.
- Diarrhea is one of the signs or symptoms of human monkeypox: Yes, No.
- -Lymphadenopathy (swollen lymph nodes) is one clinical sign or symptom that could be used to differentiate monkeypox and smallpox cases: Yes, No.
- -One management option for monkeypox patients who are symptomatic is to use paracetamol: Yes, No.
- -Antivirals are required in the management of human monkepox patients: Yes, No.
- -Antibiotics are required in the management of human monkepox patients: Yes, No.
- -People who got chickenpox vaccine are immunized against monkeypox: Yes, No.
- -There is a specific vaccine for monkeypox: Yes, No.
- -There is a specific treatment for monkeypox: Yes, No.

Attitudes of Monkeypox questions:

- I am confident that the world's population can control the monkepox worldwide: (Strongly Disagree, Disagree, Neutral, Agree, Strongly Agree).
- I am confident that the Saudi MOH and local population can control the monkepox locally: (Strongly Disagree, Disagree, Neutral, Agree, Strongly Agree).
- I am confident that the Saudi MOH and local population can control the monkepox locally: (Strongly Disagree, Disagree, Neutral, Agree, Strongly Agree).
- I have bad feelings towards monkeypox virus that it might become a worldwide pandemic: (Strongly Disagree, Disagree, Neutral, Agree, Strongly Agree).

- I think that monkepox can add new burden on healthcare system of the affected countries: (Strongly Disagree, Disagree, Neutral, Agree, Strongly Agree).
- I think that monkepox can be transmitted to Saudi Arabia: (Strongly Disagree, Disagree, Neutral, Agree, Strongly Agree).
- I think that mass media coverage about monkepox may have influence on its worldwide prevention: (Strongly Disagree, Disagree, Neutral, Agree, Strongly Agree).
- I am interested in learning more about monkepox: (Strongly Disagree, Disagree, Neutral, Agree, Strongly Agree).
- I am interested to learn more about epidemiology of the new emerging diseases: (Strongly Disagree, Disagree, Neutral, Agree, Strongly Agree).
- I am interested in learning more about Travel Medicine: (Strongly Disagree, Disagree, Neutral, Agree, Strongly Agree).
- I think that it is dangerous to travel to the countries epidemic with monkeypox: (Strongly Disagree, Disagree, Neutral, Agree, Strongly Agree).
